# PEEP, *p*-values, and pulmonary mechanics; don’t throw the baby out with the bathwater

**DOI:** 10.1186/s13054-022-04183-x

**Published:** 2022-10-10

**Authors:** Matthew E. Cove, Michael R. Pinsky, John J. Marini

**Affiliations:** 1 Department of Medicine, National University Singapore, NUHS, Tower Block Level 10, 1E Kent Ridge Road, Singapore, 119228 Singapore; 2 Department of Critical Care Medicine, University of Pittsburgh, 638 Scaife Hall3550 Terrace Street, Pittsburgh, PA 15261 USA; 3 Pulmonary and Critical Care Medicine, Regions Hospital and University of Minnesota, Minneapolis/St.Paul, MN USA

We read with interest the viewpoint by Grieco and colleagues “Why compliance and driving pressure may be inappropriate targets for PEEP setting during ARDS” [1], written in response to our viewpoint, “Are we ready to think differently about PEEP?” [2]. We are delighted our article achieved its main objectives: generating attention to the urgent need to identify a physiology-based personalized PEEP strategy and highlighting the pitfalls of titrating PEEP based only on oxygenation measures. As we suggested, PEEP selection may be guided by seeking optimal compliance, simultaneously identifying least driving pressure for a given tidal volume, a measurement associated with important outcomes in acute respiratory distress syndrome (ARDS) [3].

We thank Greico et al*.* for articulating the imperfections of compliance measurements and agree clinicians need to be cognizant that most methods estimate average compliance and fail to account for regional variation of lung tissue. We also thank them for drawing attention to the Alveolar Recruitment for ARDS Trial, which demonstrated it is unwise to couple extraordinary recruitment maneuvers with a strategy of setting PEEP 2 cmH_2_O higher than the PEEP at optimal compliance, quite different to the strategy we proposed [4]. However, we respectfully disagree that the data they presented are sufficient to reject a role of compliance in determining optimal PEEP. Greico et al. argue that selecting PEEP based on optimal compliance is unreliable by re-analyzing data from alveolar derecruitment maneuvers in 30 patients with COVID-19 [1]. In their study, individuals on volume control ventilation are transitioned from a PEEP of 5 cmH_2_O to a PEEP of 15 cmH_2_O for 30 min. Alveolar recruitment is estimated by reducing PEEP back to 5 cmH_2_O and comparing the volume of expired air to the set inspired volume during the first breath at the new PEEP.

Using this method, they demonstrated most patients experienced lung recruitment when transitioning from a PEEP of 5 to 15 cmH_2_O, regardless of whether their compliance was increased, unchanged or decreased at the higher PEEP level [1]. They suggest we cannot use compliance to seek optimal PEEP because the average recruited volume is not statistically significantly different across the three compliance groups, despite a clear trend. However, we highlight two areas of concern when interpreting their data in this way. First, drawing sweeping conclusions from a p-value in a small dataset requires extreme caution. The median recruited volume is highest in patients experiencing increased compliance and lowest in those with reduced compliance [1]. The authors conclude there is no difference because the p-value is > 0.05, but in doing so they have not considered the likelihood of a type II error in this small sample with uneven group sizes.

Second, and most importantly, an alveolar derecruitment maneuver is very different to a PEEP titration, because PEEP is changed from 5 to 15, and back again, in a single step. The authors have made the error of assuming that compliance changes in a linear fashion, in one direction, between two extremely different PEEP levels. However, when PEEP is incrementally changed in a series of small steps, this is rarely the case [5]. Since all the patients in Greico et al*.* analysis had ARDS, a PEEP of 5 cmH_2_O was probably too low for most. In those patients whose compliance was unchanged or lower at a PEEP of 15 cmH_2_O, we cannot exclude the possibility that their optimal compliance was between a PEEP of 5 and 15 cmH_2_O (Fig. 1), as seen previously in PEEP titration studies. For these patients, a PEEP of 15 cmH_2_O exceeds optimal PEEP and compliance. However, exceeding optimal PEEP does not result in loss of the recruited volume, instead, overdistention of the successfully recruited lung units may occur, reducing compliance.

**Fig. 1:**
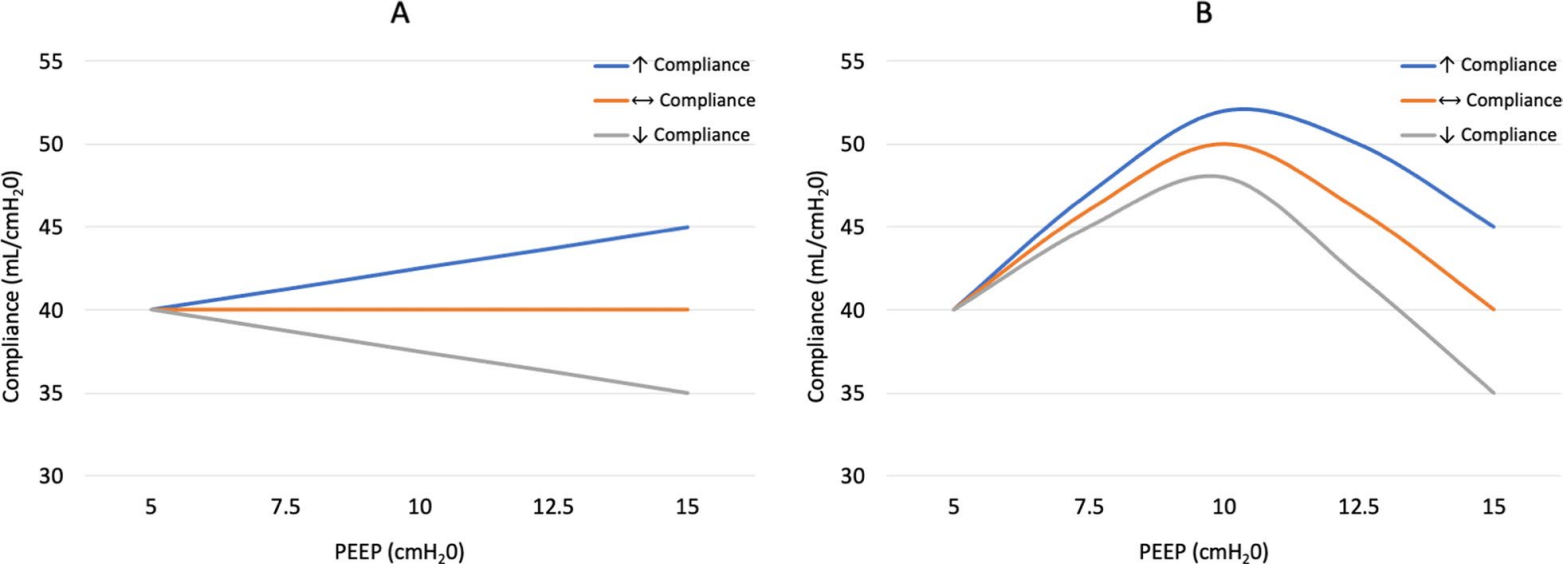
Graph A illustrates the assumption made by Greico et al., that compliance changes in a linear fashion when PEEP is incrementally changed from 5 to 15 cmH_2_O. In graph B, we modeled compliance changes expected during a PEEP titration. Using compliance reported for Greico et al.’s cohort, and PEEP titration data in ARDS patients where optimal compliance occurs at a PEEP between 9 and 12 cm H_2_O for most patients, we have modeled potential changes in compliance for patients in each of the three groups as PEEP is titrated from 5 to 15 cm H_2_O. The blue line represents patients whose compliance is increased at a PEEP of 15 cmH_2_O, the orange represents patients whose compliance is unchanged, and the gray line represents patients whose compliance is reduced at the higher PEEP level

We agree that titrating PEEP using any single physiological measure, like compliance, is imperfect. As we stated in our viewpoint, it is desirable to monitor other measures such as ventilation ratio, and we referenced work that recommends additional monitoring tools when PEEP levels are particularly high. Nonetheless, as the classical study of Suter and colleagues indicated, optimized oxygen delivery and dead space often accompany a “best compliance-determined” PEEP value [5]. Finally, we take this opportunity to highlight that using compliance to seek optimal PEEP raises many important questions, such as: How long, or how many breaths should we allow before being confident compliance has stabilized at a new PEEP? Does it matter whether PEEP is titrated upwards or downwards? Should oxygenation measures be used in combination? Should we also monitor stroke volume and cardiac output? And when, or in whom should we assess chest wall compliance? However, these are not reasons to reject our proposed strategy; rather, they should encourage us to seek better answers through new research. Therefore, we whole heartedly agree with Grieco and colleagues that urgent research is much needed.

## Data Availability

Not applicable.
